# Tranexamic acid to prevent bleeding after endoscopic resection of large colorectal polyps: a pilot project

**DOI:** 10.1093/jcag/gwae038

**Published:** 2024-10-20

**Authors:** Mandip Rai, Mary Sedarous, Connie Taylor, Jackie McKay, Lawrence Hookey, Robert Bechara

**Affiliations:** Division of Gastroenterology, Department of Medicine, Queen’s University, Kingston K7L 5G2, ON, Canada; Division of Gastroenterology, Department of Medicine, Queen’s University, Kingston K7L 5G2, ON, Canada; Division of Gastroenterology, Department of Medicine, Queen’s University, Kingston K7L 5G2, ON, Canada; Division of Gastroenterology, Department of Medicine, Queen’s University, Kingston K7L 5G2, ON, Canada; Division of Gastroenterology, Department of Medicine, Queen’s University, Kingston K7L 5G2, ON, Canada; Division of Gastroenterology, Department of Medicine, Queen’s University, Kingston K7L 5G2, ON, Canada

**Keywords:** post-polypectomy bleeding, tranexamic acid, endoscopic mucosal resection

## Abstract

**Background and aims:**

Delayed post-polypectomy bleeding (DPPB) can occur up to a month following the procedure but is typically seen within the first week. Tranexamic acid (TXA) is a member of a class of drugs called antifibrinolytic agents. It reduces fibrinolysis by slowing down the conversion of plasminogen to plasmin, which may prevent bleeding. The goal of this pilot study is to assess the feasibility of using tranexamic acid after endoscopic mucosal resection (EMR) of large (≥2 cm) non-pedunculated colorectal polyps (LNPCPs) to prevent DPPB.

**Methods:**

This was a single centre feasibility study conducted at the Kingston Health Sciences Centre in 2021. After the polypectomy was completed, IV tranexamic acid was given [1 gram of TXA in 100 mL of normal saline] and infused over a 10-min interval. The participants received tranexamic acid 1 gram PO TID to be taken for 5 days.

**Results:**

A total of 25 patients were enrolled with a mean polyp size of 3 cm. Intraprocedural bleeding occurred in 7 patients (28%) and all of these were treated with soft coagulation. Two patients had clipping for suspected muscle injury. All 25 patients received IV TXA post-procedure. Sixteen patients (64%) took every dose of the prescribed pills. One patient presented with post-polypectomy bleeding. All patients completed the day 30 follow-up phone call. There were no major adverse events.

**Conclusions:**

TXA to prevent delayed post-polypectomy bleeding (DPPB) was feasible to use with no major adverse events reported. A randomized controlled study will be needed to see if TXA can significantly reduce DPPB.

## Background and Aims

Colonoscopy and polypectomy reduce colorectal cancer incidence and mortality but are also associated with adverse events, including bleeding, perforation, sedation-related events, and rarely death.^1,2^ Delayed post-polypectomy bleeding (DPPB) can occur up to a month following the procedure but is typically seen within the first week.^3,4^ The hypothesized aetiology of DPPB is sloughing of the eschar of a cautery-induced ulcer which exposes an underlying blood vessel, and/or spreading of the thermal energy after the procedure into a submucosal blood vessel.^5^ Post-polypectomy bleeding after endoscopic mucosal resection (EMR) of large colorectal polyps (≥2 cm) has an incidence of 2.6%–9.7%.^6,7^

Guidelines now recommend prophylactic endoscopic clip closure of the mucosal defect after EMR of large non-pedunculated adenomatous polyps (LNPCPs).^8^ Multiple studies have shown benefits for prophylactic clip closure of large polyps in the proximal colon.^4,9^ Unfortunately, complete closure of defects is not possible in 43% of cases due to large size or poor accessibility^10^; and incomplete closure of the mucosal defect had been shown to be an independent predictor of delayed bleeding after EMR.^3^ This is a major issue as larger polyps are more likely to have DPPB.^3^ However, a more recent individual patient meta-analysis of over 1200 randomized trial patients showed even partial clip closure significantly reduced the risk of bleeding for proximal LNPCPs.^11^ Other modalities of prevention of DPPB are the use of haemostatic powders and gels, although the data regarding their use is equivocal.^12^ Furthermore, the novel use of sutures and endoloop to close defects for the prevention of DPPB requires more investigation regarding their efficacy. Additional considerations to this approach include the cost, technical expertise, and increased procedure time that these techniques require.

Tranexamic acid (TXA) is a member of a class of antifibrinolytic medications. It reduces fibrinolysis by slowing down the conversion of plasminogen to plasmin, which may prevent bleeding. Large studies, including randomized trials with 50 000 patients looking at the administration of TXA in trauma patients and patients with post-partum haemorrhage, have shown that it reduces bleeding and mortality.^13,14^ These large studies have also shown there is no increase in thromboembolic events, suggesting it is a safe option.

TXA has been studied previously in gastrointestinal bleeding. While 2 large studies also showed no difference in adverse events or thromboembolic events, the HALT-IT trial demonstrated an increase in deep vein thrombosis in patients on TXA.^15–17^ A key difference between our patient population and these studies is that they focussed on active acute gastrointestinal bleeding, which occurs by different etiologic mechanisms than delayed bleeding, and are generally a more unwell hospitalized population compared to routine elective polypectomy procedures.^15–17^ In contrast, our proposed study has the purpose of bleeding prevention. Additionally, as DPPB is thought to be due to sloughing of the eschar of a cautery-induced ulcer, tranexamic acid may prevent or slow the breakdown of this eschar blood clot and prevent bleeding.

Our study is the first to examine the use of TXA to prevent DPPB. There are multiple reasons why TXA would be the ideal intervention to prevent DPPB. First, it is relatively inexpensive compared to multiple endoscopic clips. Next, TXA is very simple and quick to administer. The 1 gram IV can be added to 100 mL of normal saline (NS) and given over 10 minutes. Patients undergoing colonoscopy already have an intravenous line in place for sedation that can facilitate the administration of TXA.

The goal of this pilot study is to assess the feasibility of using tranexamic acid after EMR of large (≥2 cm) non-pedunculated colorectal polyps (LNPCPs) to prevent DPPB. Results from the pilot study will assess the feasibility of conducting a future RCT.

## Methods

### Study design and setting

This was a single centre feasibility study conducted at the Kingston Health Sciences Centre (a tertiary care academic centre) from March 2021 to September 2021. The operators and patients were not blinded to the treatment. Two operators (RB and LH) performed all procedures.

Our inclusion criteria were patients aged 18–89 who have non-pedunculated colorectal polyps, polyps ≥2 cm removed by EMR (endoscopic mucosal resection) who agreed to be followed up by phone.

We excluded patients who have inflammatory bowel disease, bleeding disorder (INR ≥ 1.5 and platelets <50), ulcerated morphology of polyps or those with proven invasive cancer, patients with a higher risk of thromboembolic events (atrial fibrillation on anticoagulation, history of stroke, TIA, pulmonary embolism, deep vein thrombosis, hypercoagulable state, mechanical heart valve on anticoagulation, myocardial infarction in the last 12 months), unable to provide follow-up, unable to provide consent, pregnant patients, and patients undergoing ESD (endoscopic submucosal dissection).

### Recruitment and study intervention

Two groups of patients were approached for consideration of inclusion: patients referred to our therapeutic endoscopists for removal of a ≥2 cm LNPCP, and patients who were referred for a positive faecal immunochemical test as they are at a higher risk to have larger polyps that would meet inclusion criteria.

If the patient was a candidate for the study, the study coordinator approached the patient after they had arrived for their procedure. If the patient was interested, written informed consent was obtained before the procedure.

After obtaining consent, demographics were recorded. Participants received instructions regarding how to take their post-procedure oral medications and how to complete the study diary. A set of written instructions to take home were also provided.

The colonoscopy procedure followed standard practice for patient preparation, including placement of an intravenous (IV) line, and sedation in the form of midazolam and fentanyl.

Polyps were measured by aligning a snare of known size with the polyp to confirm they meet inclusion criteria. As per standard practice, submucosal injection with saline and/or methylene blue was performed to lift the polyp before polypectomy. “Endocut” mode was used as the electrocautery setting for all polypectomies. Coagulation of submucosal vessels after polypectomy by snare tip coagulation or forceps was performed if thought necessary by the endoscopist. Clipping could be performed only where there was concern for perforation. Intraprocedural bleeding was recorded and managed at the discretion of the endoscopist.

Immediately after the polypectomy was completed, the IV TXA was prepared by a research nurse [1 gram of TXA in 100 mL of NS] and infused over a 10 -minute interval.

Post-procedure, dispensation of oral TXA tablets was performed by the study coordinator. The participants received TXA 1 -gram PO TID to be taken for 5 days after the procedure at 0900, 1500, and 2100 hours. Participants were given a study diary to document date, time, and ingestion of the medication.

At post-procedure days 5, 14, and 30 phone calls were conducted with participants to monitor study drug compliance and adverse events. The coordinator asked participants a set of pre-determined questions to assess DPPB and potential adverse effects. Upon completion of the course of oral medication, participants mailed their completed study drug log to the study coordinator.

### Statistics and sample size

By design, this pilot study is not powered to determine the relative benefit of TXA versus placebo in the prevention of DPPB. This important scientific question will be answered by a larger trial. This pilot study was completed on 25 patients to estimate who would meet our feasibility criteria as well as to assess the study flow and process. Data are presented as frequencies and percentages and means (± standard deviation). All calculations were conducted in SPSS v28 (IBM).

### Pilot study outcomes and definitions are defined as follows

1. Study drug compliance rates, defined as 92% (23/25) of patients must receive the first dose of the study drug within 2 hours of polyp removal and 80% (20/25) of patients receiving every scheduled dose of the study drug;
2. Recruitment rates, defined as at least 60% of all eligible patients are successfully recruited;
3. Follow-up rates are defined as complete follow-up in at least 92% (23/25) of all enrolled subjects.

### Secondary outcomes measurements

1. Major adverse events: thromboembolic events, perforation requiring surgical management, post-polypectomy electrocoagulation syndrome, seizure activity, hypersensitivity reactions, CNS depression, ureteral obstruction, and vision changes;
2. Post-procedure bleeding is defined as a severe bleeding event that requires hospitalization, transfusion, colonoscopy, surgery, or another invasive intervention within 30 days after completion of the colonoscopy with polypectomy.

### Study ethics and registration

No industry-related funding was received to support this study or compensate study investigators. Funding was received from the Queen’s University Department of Medicine Research award in the clinical innovation category. The study protocol was approved by the health sciences research ethics board at Queen’s University (DMED 2372-20). Approval was also obtained from Health Canada (September 9, 2022). All patients voluntarily consented during their clinic visit by a dedicated research coordinator. The study is registered on clinicaltrials.gov NCT04559880 (September 23, 2020).

## Results

A total of 25 patients were enrolled during the study period with a median age of 64. Baseline patient and polyp characteristics are presented in Table 1. Two patients (8%) were on aspirin with the remainder on no antiplatelet or anticoagulant medication. Ninety percent of eligible patients approached consented to be in the study.

**Table 1.** Baseline patient and polyp characteristics.

Total patients	25 (12 male:13 female)
Age, years, median (range)	64 (51-87)
Aspirin use, *n* (%)	2 (8%)
Plavix use, *n*	0
Location of polyp:	
Ascending colon, *n* (%)	12 (48)
Caecum, *n* (%)	5 (20)
Hepatic flexure, *n* (%)	3 (12)
Sigmoid, *n* (%)	2 (8)
Rectum, *n* (%)	2 (8)
Transverse colon, *n* (%)	1 (4)
Size of polyp (cm), median (range)	3 (2-6)
Morphology	
Paris 2a, *n* (%)	13 (52)
Paris 1s, *n* (%)	8 (32)
Paris 1s and 2a, *n* (%)	4 (16)
Histology	
TA, *n* (%)	9 (36)
SSL, *n* (%)	8 (32)
TVA, *n* (%)	5 (20)
TVA with HGD, *n* (%)	3 (12)

The median polyp size was 3 cm (range: 2–6 cm) with the majority of polyps located in the right colon (68%). Nine polyps (36%) had histology of tubular adenoma, 8 (32%) were tubulovillous adenomas and 8 (32%) were sessile serrated lesions.

Procedure details are presented in Table 2. Intraprocedural bleeding occurred in 7 patients (28%) and all of these were treated with soft coagulation. Twopatients had clipping for suspected muscle injury. The entire defects were closed. All 25 patients received IV TXA post-procedure. Sixteen patients (64%) took every dose of the prescribed pills. Twenty one patients (84%) took at least 80% of the prescribed TXA pills.

**Table 2.** Procedure details include intraprocedural bleeding, use of clipping, and compliance with IV TXA post-procedure.

Intraprocedural bleeding, n (%)	7 (28)
Management of intraprocedural bleeding:	
Soft coagulation, *n* (%)	7 (28)
Clipping for hemostasis, *n*	0
Clipping for muscle injury, *n* (%)	2 (8)
Received IV TXA post-procedure, *n* (%)	25 (100)

One patient presented with delayed post-polypectomy bleeding. This was an 87-year-old female who was referred for resection of a large polyp after a colonoscopy was performed elsewhere for a positive FIT test. She was on low-dose aspirin. A 5 cm Paris 1s polyp was removed from the ascending colon. Final pathology showed TVA with HGD. This patient had intraprocedural bleeding that was treated with snare tip coagulation. She presented with acute DPPB the day after completing her oral TXA treatment and was admitted to the hospital. She was given a bowel prep overnight and a colonoscopy was performed the next day. No active bleeding was seen. There were several visible vessels in the defect site treated with soft coagulation. She was monitored for 3 more days in hospital and then sent home.

All patients completed the day 30 follow-up phone call. There were no major adverse events.

## Conclusions

To the best of our knowledge, this is the first pilot study assessing the feasibility of using TXA to prevent DPPB after EMR of large non-pedunculated colorectal polyps. TXA to prevent DPPB was feasible to use with no major adverse events reported. All patients received IV TXA post-procedure and completed 30 day follow-up. However, only 64% of patients took every scheduled dose of medication. 44% of patients experienced mild side effects transient side effects which may have been related to TXA including abdominal pain (20%), diarrhoea (16%), dizziness (12%), nausea (8%), cramping (8%), headache (8%) and fatigue (8%). Two patients (8%) discontinued the medication early due to side effects (one patient due to diarrhoea and one patient due to dizziness).

DPPB bleeding has a significant impact on the life of the patient as it can require hospitalization, transfusions, repeat colonoscopy, and rarely death. Additionally, the treatment of DPPB is a substantial cost to the health care system. Research efforts to date have primarily focused on the use of endoscopic clipping to prevent DPPB. The benefit of prophylactic clip closure of large polyps in the proximal colon has been shown and now is standard of care.^8^ However, endoscopic closure is technically not possible for a significant number of large mucosal defects. However, even partial closure has been shown to have a benefit.^11^ Other limitations include the time required for clip closure and cost depending on jurisdiction. Other modalities are therefore needed to prevent DPPB.

If shown to be effective, TXA could be an ideal intervention for the prevention of DPPB. It is cost-effective. Moreover, TXA can be administered quickly and simply. As the infusion only requires 10 minutes, further recovery time is not required for the patient.

There are several limitations to the study. As it is a pilot study no conclusions can be made whether TXA is effective in preventing DPPB. A randomized controlled study will be needed to see if TXA can significantly reduce DPPB. Although TXA use was shown to be safe in this study, patients at higher risk for thromboembolic events were excluded and these are arguably the higher-risk patients for DPPB. Only 64% of patients took every scheduled dose of medication despite being called the day after the procedure. In the real world without this reminder phone call, the number of compliant patients can be expected to be even lower.

Since the completion of this study, it has now become standard of care to try and clip close defects after EMR of proximal LNPCPs. Future trials would need to consider this as it may be unethical to leave proximal LNPCPs unclipped. Any future trial would require mandated attempted clip closure for proximal LNPCPs.

Eight patients had SSLs removed via EMR. Guidelines now recommend the removal of SSLs via cold snare EMR regardless of size when dysplasia is not suspected. This would dramatically lower DPPB. This would need to be considered in the design of any future trial.

The optimal length of treatment is not clear. Although DPPB is typically seen within the first week, it can occur up to a few weeks after the procedure. In this study, patients received TXA only for 5 days after the procedure. Future studies would need to consider a longer course. However, interventions that may assist in increasing compliance would need to be considered. The one patient who had DPPB in this study presented the day after completing her oral TXA treatment.

In conclusion, in this pilot study TXA to prevent post-polypectomy delayed bleeding was feasible to use with no major adverse events reported. A randomized controlled study will be needed to see if TXA can significantly reduce DPPB.

## Supplementary material

Supplementary material is available at *Journal of the Canadian Association of Gastroenterology* online.

gwae038_suppl_Supplementary_Coi_Forms

## Data Availability

The data underlying this article cannot be shared publicly due to the privacy of individuals who participated in the study. The data will be shared on reasonable request to the corresponding author.
